# Effect of healthcare system reforms on job satisfaction among village clinic doctors in China

**DOI:** 10.1186/s12960-021-00650-8

**Published:** 2021-09-08

**Authors:** Zhongming Chen, Lifang Zhou, Haiyuan Lv, Kui Sun, Hongwei Guo, Jinwei Hu, Qianqian Yu, Dongmei Huang, Dongping Ma, Zhiqiang Feng, Changhai Tang, Mengna Dai, Wenqiang Yin

**Affiliations:** 1 School of Management Weifang Medical University, NO. 7166, Western Baotong Road, Weifang, Shandong China; 2 School of Public Health Weifang Medical University, NO. 7166, Western Baotong Road, Weifang, Shandong China

**Keywords:** New health care system reform, Village clinic doctors, Job satisfaction

## Abstract

**Background:**

Village clinic doctors (VCDs) are part of the health service force in rural China. VCDs’ job satisfaction (JS) is important to the stability of the three-tiered health service system. Since 2009, the Chinese government launched a new health care system reform (NHCSR) which affected VCDs significantly. This study aimed to analysing the effect of NHCSR on JS among VCDs.

**Methods:**

All the data came from three surveys in Shandong Province conducted in 2012, 2015 and 2018. In 2012, an originally designed questionnaire was used to conduct a baseline survey of 405 VCDs from 27 townships in nine counties. In 2015 and 2018, 519 and 223 VCDs in the same counties were surveyed with the same questionnaire. Descriptive analysis and ANOVA were used to analyse the level and changes in VCDs’ JS.

**Results:**

The mean scores of VCDs’ total JS were 2.664 ± 1.069, 3.121 ± 0.931 and 2.676 ± 1.044 in 2012, 2015 and 2018, respectively, with a significant difference (*F* = 28.732, *P* < 0.001). The mean scores of the medical practice environment and the job itself showed a continuous downward trend. The trends of the mean scores for job reward, internal work environment and organizational management were consistent with the trend for total JS.

**Conclusion:**

The NHCSR had a partly negative impact on VCDs’ JS. Policy-makers should pay more attention to VCDs’ job reward and medical practice environment. With the implementation of new reform policies, VCDs’ JS should be the subject of more systematic and detailed research.

## Background

As an important indicator of human resource management, job satisfaction (JS) refers to the positive emotional state of employees regarding their work or work experience [1]. Previous studies [2–5] showed that doctors’ JS was an important factor affecting health service quality, patient satisfaction and the stability of the doctors’ jobs and the health system over all. Village clinic doctors (VCDs) are health workers rooted in rural China, originating from the “barefoot doctors” in the early days of the founding of the People’s Republic of China. They are the cornerstone of the three-tiered health service system. The three-tiered health service system is composed of health service institutions in county, township and village, including county hospitals, township hospitals, village clinics and so on. Its function is to provide primary health care services for rural residents. Village clinics are the cornerstone of the three-tiered health service system. As the main staff of village clinics, VCDs have played an important role in ensuring the health of rural residents for a long time. They are also known as the health protectors of rural residents [6]. According to the statistics of the National Health Commission of the People’s Republic of China, since 2015, the annual number of visits to village clinics has amounted to more than 1.6 billion, accounting for more than 37% of the visits to primary medical institutions [7]. A high level of JS among VCDs is conducive to providing better medical services for rural patients and supporting the stability and sustainable development of the health service system [8, 9].

Inadequate health resources and health service capacity at the primary level (especially in rural areas) have been problems in China’s health system, directly leading to and aggravating the difficulty and expense of medical treatment. To effectively solve these problems, the Chinese government launched the new health care system reform (NHCSR) in 2009, which adheres to the basic principles of “ensuring the basics, strengthening the locals and constructing the mechanisms”. During the first phase of the reform (2009–2011), the focus of the reform was on constructing a basic medical service system, basic public health service system, basic medical insurance and essential medicine system to strengthen the capacity of primary health services [10]. In 2015, the State Council put forward the implementation of the grading healthcare system [11], aiming to improve the quality of primary health services and attract patients to primary health institutions to promote the formation of a reasonable order of diagnosis and treatment. In 2018, the Central Committee of the Communist Party of China put forward the “Rural Revitalization Strategy” [12], which calls for “healthy rural” construction and proposes higher requirements for village-level health services. Undoubtedly, VCDs have been the focus of the NHCSR. The NHCSR has changed VCDs’ service function and main source of income. After the implementation of the NHCSR, VCDs have to provide essential medical and public health services for rural residents, instead of only medical services. And VCDs’ main source of income has changed from market to government finance. What’s more, VCDs’ right to use drugs has been restricted. However, statistics showed that [13] from 2009 to 2013, the annual number of visits to village clinics increased from 1.552 billion to 2.012 billion. However, by 2018, the annual number of visits to village clinics had dropped to 1.67 billion, and the proportion of visits to clinics had dropped to 20.1% from 27.51% (2013-year). The limitation of drug use and medical insurance reimbursement, imperfect incentive mechanism and the decline of VCDs’ JS and enthusiasm are potential reasons for this phenomenon [14, 15].

Since the NHCSR was implemented, VCDs have received increasing attention among health management researchers. Previous studies [16–22] covered aspects such as VCDs’ service capacity, team building, education and training, income treatment, professional mentality, incentive and restraint mechanisms, recognition and evaluation of the NHCSR. Job satisfaction was defined as having a positive emotional state resulting from a person’s appraisal of his or her job [23]. It was an important topic in this research area. Zhang et al. [24] studied the current level of the JS of VCDs in western China, and the results showed that 44.6% of VCDs expressed dissatisfaction with their work. Zhang et al. [25] described the current situation of VCDs’ JS in Jiangxi Province, and the results showed that 87.28% of VCDs expressed dissatisfaction. Ding et al. [26] described the current JS of VCDs in Nanjing, and the results showed that 46.5% of VCDs were dissatisfied with their job. Sun et al. [27] described the income satisfaction of VCDs, and the results showed that 29.9% of VCDs were not satisfied with the current monthly average income level. Chen et al. [28] studied the training satisfaction of VCDs, and the results showed that 86.47% of VCDs were satisfied with the current professional training. Yang analysed gender differences in the JS of VCDs in western China. The results showed that the JS of male VCDs was 3.16 ± 0.74 and that of female VCDs was 3.22 ± 0.66 [29].

Most previous studies were cross-sectional studies conducted at a certain moment in time. These studies showed that the current level of VCDs’ JS is low. Previous studies provided reference and good basis for the design and analysis of this study. Although most of the studies discuss the impact of the NHCSR on VCDs’ JS, they cannot directly reflect the changes in the JS of VCDs since the implementation of the NHCSR. With the data of three surveys on VCDs conducted in Shandong Province in 2012, 2015 and 2018, this study analysed the changes in the JS of VCDs since the NHCSR and discussed the possible reasons for these changes from the perspective of the NHCSR. The results of this study provide a basis and reference for improving the JS and stability of VCDs.

## Methods

### Study design and sampling

Subjects of this study are VCDs. Shandong Province is a province in eastern China. In 2019, Shandong Province had 16 prefecture-level cities (an administrative level below the province and above the county; there were 17 prefecture-level cities in and before 2018), a population of 100.70 million (including a rural population of 38.76 million) and a per capita GDP of 70,653 yuan and belonged to the economically developed regions of China. In 2018, there were 53,246 village clinics and 96,253 VCDs in Shandong Province. On average, there were 1.8 VCDs in each village clinic.

All the data in this study came from three surveys of VCDs held in Shandong Province in 2012, 2015 and 2018, with 405, 519 and 223 valid questionnaires and response rates of 92.9%, 94.3% and 92.9%, respectively. In 2012, we conducted a baseline survey using a multistage sampling method. First, according to the economic level, three prefecture-level cities, Jinan, Linyi and Dezhou, were selected from 17 prefecture-level cities in Shandong Province, which represent economically developed areas, moderately developed areas and underdeveloped areas, respectively. Then, three counties with different economic levels were randomly selected from each prefecture-level city, three townships were randomly selected from each county, and one village clinic doctor was randomly selected from each village clinic in the three townships to fill in the questionnaire in the township health centre. In 2015 and 2018, follow-up surveys were conducted, but thanks to urbanization and regional health development in recent years, some village clinics were abolished or merged and could not be traced. Coupled with the demission of VCDs [30, 31], the number of VCDs decreased, resulting in a larger reduction in the sample size in 2018. To ensure that the privacy of the respondents’ information was effectively protected, the survey was carried out anonymously by a self-administered questionnaire. All the respondents were fully informed of the value and significance of the survey to improve the response rate. All investigators were trained intensively before the investigation to ensure survey quality. In the investigation, one investigator was responsible for reviewing the quality of the questionnaire specially. If the questionnaire was found to be problematic, the respondents were invited to supplement and improve it on the spot. After completing the survey, another investigator reviewed the questionnaire again and eliminated the questionnaires with poor quality.

### Measurement instruments

The Chinese Physicians’ Job Satisfaction Questionnaire compiled by Yin et al. was used to measure the JS of VCDs [32, 33]. It included satisfaction with the job itself (such as job significance, professional interest and job value), satisfaction with job rewards (such as income level, rationality of income distribution, opportunities for further study and promotion of professional titles), satisfaction with the internal working environment (such as office environment, equipment resources, colleague relationships), satisfaction with the medical practice environment (such as doctor–patient relationships, social recognition), and satisfaction with organizational management (such as management system, leadership behaviour, identification with the organization). All the items were rated with a five-point Likert scale, ranging from 1 (very dissatisfied) to 5 (very satisfied). The medical practice environment is a negative item, so it is reverse scored. This questionnaire has been used in many studies and has been verified to have good reliability and validity [34–36].

### Statistical analysis

First, descriptive analysis was conducted on the demographic characteristics and JS of VCDs. Then, *χ*^2^ test, one-way ANOVA and post hoc least-significant difference (LSD) were adopted to compare the differences in JS among groups. The significance level of all tests was set at *P* < 0.05 (two-tailed).

## Results

### Demographic characteristics

The demographic characteristics of the participants in the three surveys in 2012, 2015 and 2018 are shown in Table 1. In the three surveys, most VCDs were married male with secondary vocational school diploma, who were between the ages of 31 and 40. At the same time, most of them worked in the village clinics that participated in the integrated management led by township health centres, and have the qualification of rural doctors—a qualification formulated by the Chinese health administrative departments for medical personnel working in village clinics, it requires lower competence than that of licensed assistant doctors and its scope of practice is general medicine.

**Table 1:** Demographic characteristics of VCDs in three surveys

Characteristics	2012 (*N* = 405)	2015 (*N* = 519)	2018 (*N* = 223)	*χ*^2^	*P*
	*N* (%)	*N* (%)	*N* (%)		
Gender					
Male	245 (60.5)	342 (65.9)	152 (68.2)	4.579	0.101
Female	160 (39.5)	177 (34.1)	71 (31.8)		
Age					
≤ 30	33 (8.3)	28 (5.4)	9 (4.0)	10.441	0.107
31~	159 (39.8)	207 (39.9)	81 (36.3)		
41~	109 (27.3)	171 (32.9)	80 (35.9)		
51~	99 (24.8)	113 (21.8)	53 (23.8)		
Marital status					
Unmarried/widowed/divorced	16 (4.0)	21 (4.1)	4 (1.8)	3.537	0.171
Married	389 (96.0)	496 (95.9)	219 (98.2)		
Educational background					
No medical educational background	35 (8.7)	39 (7.6)	10 (4.5)	6.797	0.147
Secondary technical school graduates	305 (75.5)	386 (75.2)	163 (73.4)		
Junior college degree or higher	64 (15.8)	88 (17.2)	49 (22.1)		
Practice qualification					
Medical practitioner	112 (30.7)	34 (6.6)	21 (9.5)	139.627	< 0.001
Assistant medical practitioner	50 (13.7)	33 (6.4)	40 (18.2)		
Practice qualifications of rural doctors	203 (55.6)	452 (87.1)	159 (72.3)		
Integrated care					
Yes	344 (87.8)	495 (96.1)	218 (97.8)	33.932	< 0.001
No	48 (12.2)	20 (3.9)	5 (2.2)		

Percentage of VCDs with missing data on age (1.2% in 2012), marital status (0.4% in 2015), educational background (0.2% in 2012, 1.2% in 2015 and 0.4% in 2018), practice qualification (9.9% in 2012 and 1.3% in 2018), and integrated care (3.2% in 2012 and 0.7% in 2015)

### Job satisfaction comparison

The results of VCDs’ JS across the three surveys are shown in Table 2. First, we consider the change in the job itself satisfaction score. According to the results of one-way ANOVA, the mean score of VCDs’ job itself satisfaction showed a downward trend (*F* = 113.696, *P* < 0.001). According to the results of the LSD test, the scores for the satisfaction with the job itself in 2015 and 2012 were not significantly different, with values of 2.769 ± 0.741 and 2.819 ± 0.837, respectively. However, the score was significantly lower in 2018 (1.921 ± 0.715) than in 2012 and 2015.

**Table 2:** Comparison of VCDs’ JS in three surveys

Items	2012 ($\overline{{\varvec{X}}}\pm {\varvec{S} }$)	2015 ($\overline{{\varvec{X}}}\pm {\varvec{S} }$)	2018 ($\overline{{\varvec{X}}}\pm {\varvec{S} }$)	*F*	*P*
Satisfaction with the job itself	2.819 ± 0.837	2.769 ± 0.741	1.921 ± 0.715	113.696	< 0.001
Job rewards satisfaction	1.991 ± 0.931	2.233 ± 0.728	1.769 ± 0.852	26.569	< 0.001
Internal work environment satisfaction	3.071 ± 0.722	3.117 ± 0.604	2.735 ± 0.762	25.630	< 0.001
Medical practice environment satisfaction	3.249 ± 0.876	2.069 ± 0.739	1.385 ± 0.626	478.539	< 0.001
Organizational management satisfaction	2.854 ± 0.906	3.168 ± 0.787	2.960 ± 0.761	15.793	< 0.001
Total JS	2.664 ± 1.069	3.121 ± 0.931	2.676±1.044	28.732	< 0.001

Second, the change in the job reward satisfaction score is considered. According to the results of one-way ANOVA, the mean score of VCDs’ job reward satisfaction first increased and then decreased (*F* = 26.569, *P* < 0.001). According to the results of the LSD test, the scores for job reward satisfaction in 2012, 2015 and 2018 were significantly different, with 1.991 ± 0.931, 2.233 ± 0.728 and 1.769 ± 0.852, respectively.

Third, the change in the internal work environment satisfaction score is considered. According to the results of one-way ANOVA, the mean score of VCDs’ internal work environment satisfaction first increased and then decreased (*F* = 25.630, *P* < 0.001). According to the results of the LSD test, the difference in internal work environment satisfaction between 2012 and 2015 was not statistically significant, with values of 3.071 ± 0.722 and 3.117 ± 0.604, respectively. However, the score was significantly lower in 2018 (2.735 ± 0.762) than in 2012 and 2015.

Fourth, the change in the score for satisfaction with the medical practice environment is considered. According to the results of the one-way ANOVA test, the mean score of VCDs’ satisfaction with the medical practice environment showed a downward trend (*F* = 478.539, *P* < 0.001). According to the results of the LSD test, the scores for satisfaction with the medical practice environment across 2012, 2015 and 2018 were significantly different, with 3.249 ± 0.876, 2.069 ± 0.739 and 1.385 ± 0.626, respectively.

Fifth, the change in the score for the satisfaction with organizational management is considered. According to the results of the one-way ANOVA test, the mean score of VCDs’ satisfaction with organizational management first increased and then decreased (*F* = 15.793, *P* < 0.001). According to the results of the LSD test, the scores for the satisfaction with organizational management between 2012 and 2018 were not significantly different, with values of 2.854 ± 0.906 and 2.960 ± 0.761, respectively. However, the score was significantly higher in 2015 (3.168 ± 0.787) than in 2012 and 2018.

Finally, the change in the total JS score is considered. The results showed that the mean score of VCDs’ total JS first increased and then decreased (*F* = 28.732, *P* < 0.001). According to the results of the LSD test, the scores for total JS in 2012 and 2018 were not significantly different, with values of 2.664 ± 1.069 and 2.676 ± 1.044, respectively. However, the score was significantly higher in 2015 (3.121 ± 0.931) than in 2012 and 2018.

## Discussion

After the implementation of the NHCSR, the JS of VCDs showed a trend of first rising and then falling. Specifically, satisfaction with the medical practice environment and the job itself showed a continuous downward trend. The change trends of satisfaction with job reward, internal work environment and organizational management were consistent with the trend of total JS. At the beginning of the NHCSR, VCDs were mainly dissatisfied with job rewards, while in the middle and late stages of the NHCSR, the subject of their dissatisfaction shifted to the medical practice environment.

### The total JS of VCDs showed a downward trend after the first rise

The results of the survey in 2012 showed that the mean scores of satisfactions with the medical practice environment and internal work environment were high. It was similar to the finding of Fang et al. [37]. To a certain extent, this showed that the NHCSR had achieved remarkable results at the beginning. In 2009, China started the NHCSR based on the basic principles of “ensuring the basics, strengthening the locals, constructing the mechanisms”, and 2009–2011 was the first stage of the reform. During this period, the Chinese government issued a series of policies aimed at strengthening the construction of primary health institutions and improving primary health facilities [38]. Therefore, the mean score of satisfaction with the internal work environment was higher than the scores of other aspects (except medical practice environment). This was consistent with the finding of Li et al. [39] and Xu et al. [40] found that, the improvement in the buildings and health facilities of village clinics significantly improved the recognition of village clinics among rural residents. Moreover, a series of policies to support the development of primary health institutions was implemented. As a result, the mean score of satisfaction with the medical practice environment was higher than the scores of other aspects. The results of the survey in 2015 showed that the mean score of the total JS of VCDs was higher than that in 2012. This was mainly attributed to the improvement in VCDs’ satisfaction with job rewards and organizational management. The results of the survey in 2018 showed that the mean score of the total JS of VCDs was lower than the score in 2015. The scores for four of the five dimensions of JS decreased. This phenomenon is worthy of the attention of health management researchers and policymakers. One possible reason is that the gap between the current state and the expectations of job had increased [41], such that the JS level decreased. Another possible reason is the change of the qualification of VCDs. Previous research [42] showed that, the higher the qualification, the lower the JS of VCDs. Compared with 2015, the proportion of VCDs with the qualifications of medical practitioner and assistant medical practitioner increased significantly in 2018. This may have an impact on the JS of VCDs.

### Satisfaction with job rewards, internal work environment and organizational management showed a downward trend after the first rise

The year 2015 marked the close of the 12th Five-Year Plan for China’s National Economic and Social Development (2011–2015). Over these years, government investment in primary health institutions, such as village clinics, continued to increase, leading to the optimization of the buildings and health facilities of village clinics and increasing the financial subsidies for VCDs. Moreover, the management system and security policies of primary health gradually improved [43, 44]. Therefore, the scores of the satisfaction with the three indicators improved compared with the results of the survey in 2012. However, there are many reasons for the decline in the scores for the three indicators in 2018. First, the incentive mechanism is not perfect. On the one hand, the level of matching between incentive measures and the incentive preference of village doctors is low. Problems that concern VCDs, such as professional risks, welfare and personal income, have not been effectively solved [45]. On the other hand, the connection between personal efforts and work performance is not strong enough. There is an egalitarian tendency in the granting of financial subsidies, which inhibits the enthusiasm of VCDs to some extent [46]. Second, the increase in financial subsidies for VCDs has been slow. After the implementation of the NHCSR, subsidies for the essential medicine system and basic public health services became the main sources of income for VCDs. The subsidy of the essential medicine system is the “recurrent balance of revenue and expenditure subsidy” issued by the government for government-run village clinics that implemented the essential medicine system. The amount of compensation is related to the size of the population served by the VCDs, not the actual balance of income and expenditure of village clinics. Moreover, the level of compensation is low [47, 48]. The subsidy of basic public health services is granted by the government to VCDs who provide basic public health services. It increased from 15 yuan per service population in 2011 (of which 40% were allocated to VCDs) to 69 yuan in 2019 [49, 50]. Beginning in 2014, this policy required that all the new compensation funds in rural areas be used in village clinics, which means that the current level is approximately 47 yuan per service population. The subsidy level is directly related to the population served by VCDs and affected by the hollowing of rural areas. In recent years, although the standard of financial subsidies has continuously improved, the actual financial subsidies of VCDs have not increased significantly. Third, the pressure brought by increased workload has been much higher than the sense of gain brought by the increased income for VCDs. For example, before the NHCSR, VCDs only undertook tasks related to basic medical services. However, after the implementation of the NHCSR, basic public health services were added, and the service content increased from 9 to 12 main functions [49, 50]. In addition, the increase in workload extended the part-time working hours of VCDs. Previous studies have shown that most VCDs work part-time in agricultural production, commercial activities or temporary employment [24], in addition to providing health services, to effectively provide for their families. However, with the increasing workload, VCDs have had to spend more time working to provide health services, resulting in the continuous reduction in income from their other part-time jobs. A fourth problem regards the continuous adjustment of basic public health services. The project has played a vital role in promoting health. However, the continuous adjustment of technical specifications and assessment systems has caused confusion among VCDs [51]. Fifth, there is no sustainable long-term investment mechanism for the construction of village clinics and health facilities [52]; instead, the government has most often provided one-time investment in infrastructure construction, purchasing and maintenance of health facilities of village clinics. The health facilities that received investment in the initial stage of the NHCSR have gradually aged, which makes it difficult for doctors to meet the needs of rural patients. Therefore, the limitations of health facilities are another important factor restricting VCDs from providing medical services. Sixth, the operating funds of village clinics are not guaranteed. This leads to a poor medical environment in village clinics [53]. Because all the subsidies are related to the service population, without connection to the actual burden of operating a given village clinic, VCDs have to minimize expenditure to control the operating costs of the clinic. This inevitably has a negative impact on the medical environment of the village clinics.

### Satisfaction with the medical practice environment and the job itself showed a trend of continuous decline

There are five possible reasons for the decline in satisfaction with these aspects. First, the frequent doctor–patient disputes in China in recent years have placed great psychological pressure on VCDs, which has been confirmed by the studies of Hesketh Therese, Wu et al. [54, 55]. Second, the protection mechanism for medical disputes of VCDs is imperfect. VCDs in most areas do not have medical dispute liability sharing insurance. Moreover, some VCDs who purchase medical dispute liability sharing insurance cannot be protected by the insurance because of a series of problems, such as high restrictions and little compensation [56]. The results of the survey in 2012 showed that the VCDs were most dissatisfied with job rewards, while according to the surveys of 2015 and 2018, they were most dissatisfied with the medical practice environment, and their satisfaction was lower in 2018 than in 2015. This indicated that the deteriorating medical practice environment had an increasingly serious impact on the JS of VCDs. This is basically consistent with the findings of Gan et al. [57] for general practitioners. Third, VCDs’ work autonomy is insufficient. The main manifestation of this is the limitation of drug use. Since the implementation of the NHCSR, all government-run primary health institutions have had to implement the essential medicine system. This policy limits VCDs to prescribing only essential medicines [58]. Moreover, with the advancement of the NHCSR, the implementation and supervision of this policy is becoming increasingly stringent. This regulation effectively controls the abuse of antibiotics and injections in rural areas [59, 60] but greatly restricts the autonomy of drug prescription by VCDs and weakens the medical service capacity of village clinics to some extent [14]. As a result, VCDs find it increasingly difficult to meet the requirements of their jobs. Fourth, the career prospects of VCDs are not optimistic. On the one hand, the status of most VCDs is still “semi-agricultural and semi-medical”. It is difficult for VCDs to obtain the same promotion opportunities regarding professional title as other medical staff [61]. On the other hand, because they have experience mainly with single diseases and simple conditions, young and middle-aged VCDs’ professional ability improvement is slow, so they have a low sense of achievement and personal value. Fifth, doctors face competition from private clinics and the attraction of doctors from county-level medical institutions [62].

This is the first study to monitor the JS of VCDs over time since the implementation of the NHCSR in China. Some limitations of the study should be noted. First, a self-report questionnaire was used to collect information. Social desirability effect caused by observation bias was therefore unavoidable. Second, because influencing factors were not investigated, the reasons for the changes in VCDs’ JS may not be limited to those mentioned in this paper. Third, because the places where the VCDs practised were relatively scattered and there were only 1–2 doctors in most village clinics, it was difficult to carry out a large sample follow-up survey. Influenced by the sample size, the results of this study may offer limited representativeness regarding the JS of VCDs in China.

## Conclusion

With the implementation of a healthy rural strategy and a graded health care system, VCDs have been given new and higher requirements. Analysing the JS of VCDs is an urgent requirement to improve the capacity of primary health institutions and stabilize the three-tiered health service system. What’s more, it plays an important role in implementing the principle of “strengthening the locals” in the NHCSR. The results of our analysis showed that, the NHCSR had a partly negative impact on VCDs’ JS. And the decline of VCDs’ JS may have a negative impact on the quality of health services and patients’ medical experience. In recent years, the Chinese government has launched a series of reform policies focusing on the construction of a regional medical consortium (it is a reform to improve the service efficiency of health system by integrating health resources in a region), reform of a medical insurance payment system, and reform of health management mechanism, together with the development and promotion of internet medical technology. All of these factors provide new impetus and hope for VCDs. Therefore, more systematic and detailed research should be carried out to accurately observe the impact of the NHCSR on the JS of VCDs.

## Data Availability

Data may be made available by the authors upon request.

## Abbreviations

NHCSR: New health care system reform
ANOVA: Analysis of variance
LSD: Least-significant difference
JS: Job satisfaction
VCDs: Village clinic doctors
